# Supplementary material for: WordCommentsAnalyzer: A windows software tool for qualitative research
Source: F1000Res. 2018 Sep 4;7:536. Originally published 2018 May 3. [Version 2] doi: 10.12688/f1000research.14819.2 (PMC6137411; doi:10.12688/f1000research.14819.2)
Supplement: Supplementary file 3 [file f1000research-7-17614-s0002.tgz › b2a79496-4ada-47c4-9682-a99ce867836d.zip!/Eight Iranian Health Journals/Yafteh _7.docx]

© Yafteh  . This work is licensed under CC BY 4.0 (https://creativecommons.org/licenses/by/4.0/).

Volume & Issue: دوره 18 - شماره 3

Title: بررسی اپیدمیولوژیک بیماری فنیل کتونوری در استان لرستان

http://yafte.lums.ac.ir/article-1-2349-fa.html

مقدمه: فنیل کتونوری یک بیماری متابولیک با الگوی وراثت اتوزوم مغلوب است که به علت کمبود یا فقدان آنزیم فنیل آلانین هیدروکسیلاز در کبد ایجاد می‌شود. بروز بیماری فنیل کتونوری به طور متوسط 1 از 10000 نفر است. هدف از این مطالعه تعیین ویژگی‌های اپیدمیولوژیک بیماری فنیل کتونوری در استان لرستان است. مواد و روش ­ها: با انجام یک مطالعه اپیدمیولوژیک توصیفی تمامی 81 بیمار فنیل کتونوری شناخته‌شده در استان لرستان تا اسفند 1393 مورد بررسی قرار گرفتند. بر اساس اهداف و متغیرهای مطالعه پرسشنامه‌ای طراحی شد که اطلاعات لازم جهت تکمیل آن از طریق مصاحبه با والدین و بررسی پرونده بیماران به دست آمد و یافته‌ها با نرم‌افزار  SPSS نسخه 16 و با تهیه جداول، گراف و آزمون های کای دو و تی تست تجزیه و تحلیل شد. یافته ­ها: نتایج نشان می دهد که شیوع فنیل کتونوری در استان لرستان 3/4 در 100000 نفراست. از این تعداد 20 نفر (7/24%) از طریق غربالگری و 61 نفر (3/75%) از طریق سایر روش‌ها شناسایی شدند. از این تعداد 46 نفر(8/56%) دختر و 35 نفر (2/43%) پسر بودند. تقریباً 75 درصد موارد فنیل کتونوری سابقه ازدواج فامیلی در والدین داشتند.. شیوع بیماری در نورآباد با سایر شهرستان‌ها اختلاف معنی‌داری دارد. بحث و نتیجه­ گیری: غربالگری نوزادان از نظر ابتلا به فنیل کتونوری ضروری است و بایستی تاکید بر انجام آن در روزهای  5-3 تولد باشد. درخانواده‌های دارای فرزند مبتلا بیماری، انجام آزمایش تشخیص پیش از تولد برای سایر موارد حاملگی لازم است.

Title: ارزیابی عملکرد تصفیه‌‌ خانه فاضلاب خرم‌‌آباد توسط شبکه هوش مصنوعی

http://yafte.lums.ac.ir/article-1-2350-fa.html

مقدمه: در دو دهه اخیر کاربرد مدل‌ های هوش مصنوعی به منظور بهره‌ برداری صحیح از تصفیه ‌خانه‌ و حفظ پایداری فرآیند ‌های تصفیه در شرایط مطلوب، توسط محققین بسیار فراگیر شده است. این مدل ‌ها به منظور شبیه ‌‌سازی رفتار سیستم تصفیه‌ خانه می ‌توانند به عنوان یک ابزار مؤثر برای پیش ‌بینی عملکرد تصفیه ‌خانه به کار برده شوند. هدف از انجام این مطالعه ارزیابی عملکرد تصفیه‌‌‌ خانه فاضلاب خرم‌‏آباد توسط شبکه هوش مصنوعی می ‌‏باشد. مواد و روش ­ها: در این مطالعه با استفاده از مدل ANN-LM و مبنا قرار دادن مشخصه‌ های کیفی اندازه‌‌ گیری ‌شده در ورودی تصفیه‌ خانه (T، pH،DO ، BOD، COD، TSS،TDS ، NO3، PO4)، مقدار متناظر سه مشخصهBOD ،COD  و TSS در خروجی تصفیه ‌خانه پیش‌ بینی گردید. شاخص ‌های آماری مورد استفاده شامل R، MSE و نرم ‌افزار ‌های مورد استفاده شامل Matlab و spss (آزمون آماری T-test) بودند. یافته ­ها: براساس نتایج،BOD ،COD  و TSS به ترتیب با حداکثر R، 98/0، 91/0 و 92/0 برای داده‌ های آموزش و 5/0، 66/0 و 5/0 برای داده‌ های آزمایش و حداقل MSE، 5/3، 15/33 و17/2 برای داده‌ های آموزش و 11، 115 و 99/20 برای داده‌ های آزمایش پیش ‌بینی شدند و نتایج قابل قبولی ارائه شد. همچنین، با محاسبه درصد بازده حذف آلاینده ‌‌ها در خروجی تصفیه‌ خانه مشخص شد حداکثر بهره‌‌ وری حذف در تصفیه ‌خانه مربوط به آلاینده TSS بوده و معادل 68/87 درصد است. سایر آلاینده‌ ها نیز مقادیری نزدیک به TSS داشتند. بحث و نتیجه­ گیری: در این مطالعه ANN-LM یک ابزار قابل اطمینان برای پیش ‌‌بینی عملکرد سیستم تصفیه‌ خانه فاضلاب خرم‌آباد ایجاد نمود و توانست بر مبنای پارامتر ‌های اندازه‌ گیری شده، کیفیت پساب خروجی را پیش ‌‌بینی نماید. بازده حذف آلاینده‌ ها از طریق مقادیر برآوردی با شبکه ANN-LM به گونه ‌ای بوده که به واسطه نزدیکی با مقادیر مشاهداتی مبین کارایی خوب این مدل به کار برده شده ‌‌است. همچنین تصفیه‌ خانه در کاهش مقادیر کیفی در حد مقادیر استاندارد توصیه‌ شده از سوی سازمان حفاظت محیط ‌زیست، از کارایی خوبی برخوردار است.

Title: بررسی شیوع اختلال وسواسی-جبری در بیماران وابسته به مواد مخدر مراجعه کننده به کلینیک های ترک اعتیاد وابسته به دانشگاه علوم پزشکی لرستان در سال ١٣٩٣

http://yafte.lums.ac.ir/article-1-2351-fa.html

مقدمه: وجود علایم وسواس­گونه در افراد سوءمصرف­کننده مواد و نبود مطالعه­ای منسجم در این زمینه ضرورت انجام مطالعه حاضر را نشان می­دهد. پژوهش حاضر با هدف بررسی شیوع اختلال وسواسی-جبری در بیماران وابسته به مواد مخدر مراجعه­کننده به کلینیک­های ترک اعتیاد وابسته به دانشگاه علوم پزشکی لرستان انجام شد. مواد و روش ­ها: پژوهش حاضر یک مطالعه­ی توصیفی مقطعی بود. در این پژوهش 200 نفر از بیماران وابسته به مواد مخدر مراجعه کننده به کلینیک­ های ترک اعتیاد وابسته به دانشگاه علوم پزشکی لرستان از دی ماه تا اسفند ماه سال 1393 به روش در دسترس انتخاب شدند. جمع­ آوری داده ­ها با استفاده از پرسشنامه جمعیت­ شناختی و پرسشنامه غربالگری وسواس Zohar-fineberg  انجام شد. برای تحلیل داده­ ها از شاخص­ های آمار توصیفی و استنباطی با نرم افزار SPSS-20 استفاده شد. یافته­ ها: در این مطالعه تمام شرکت­ کنندگان مرد بودند. میانگین و انحراف معیار سن و مدت مصرف مواد به ترتیب 96/11±72/41 و 87/8±02/12 بود. از این افراد 71% متأهل، 56/0% دارای شغل آزاد و 43/0% تحصیلات دیپلم داشتند. همچنین 31 نفر (5/15%) افراد با اختلال وسواسی-جبری و 169 نفر (5/84 %) بدون اختلال وسواسی-جبری بودند. بحث و نتیجه­ گیری: در ایجاد اختلال سوءمصرف مواد، اختلالات و علایم دیگر می­توانند سهم داشته و مهم باشند، بنابراین تشخیص و توجه به آن­ها بسیار مهم است. بر همین مبنا، متخصصین بالینی می­توانند براساس نتایج چنین پژوهش­هایی به طراحی کارآزمایی­ های بالینی مناسب و یا اصلاح مداخله­ های موجود اقدام نمایند.

Title: بررسی غلظت ترکیبات آلی فرار و گازهای آلاینده در مراکز چاپ و تکثیر شهر خرم آباد در سال 1394

http://yafte.lums.ac.ir/article-1-2352-fa.html

مقدمه: با توجه به تولید آلاینده های گازی و آیروسولی فراوان توسط صنعت چاپ و تکثیر، ارزیابی کیفیت هوا در داخل و خارج این مراکز در سطح شهر خرم آباد مورد بررسی قرار گرفت. مواد و روش­ ها: در یک مطالعه توصیفی، پنج آلاینده گازی از آلاینده های معیار(CO،NO ،NO2 ،SO2  وO3 ) و ترکیبات آلی فرار مورد سنجش قرار گرفت. در کل 360 نمونه از هوای داخل و خارج مراکز چاپ و تکثیر در دو فصل تابستان و زمستان گرفته شد. نمونه برداری به دو روش قرائت مستقیم و نمونه برداری محیطی انجام گرفت. در روش قرائت مستقیم از دستگاه آنالایزر محیطی برای سنجش آلاینده های معیار گازی و از دستگاه phochek5000  برای اندازه گیری VOCs استفاده گردید. در نمونه برداری محیطی از پمپ های محیطی و لوله های جاذب کربن فعال SKC به صورت تصادفی-مداوم استفاده گردید. نمونه ها با حلال دی سولفید کربن آماده سازی شدند و با دستگاه کروماتوگراف گازی مورد تجزیه قرار گرفتند. از آزمون آماری t برای آنالیز نتایج استفاده گردید. یافته ­ها: نتایج پژوهش نشان داد که در بین آلاینده های شاخص بجز گاز CO و در بین ترکیبات آلی فرار غلظت آلاینده های بنزن و تولوئن در هوای مراکز چاپ و تکثیر بیشتر از استاندارد EPA برای اماکن بسته می باشد. همچنین بین غلظت بنزن و تولوئن در فصل تابستان و زمستان اختلاف معنی داری وجود داشت (05/0P<). در همه اندازگیری ها در فصول تابستان و زمستان میزان گاز O3 در داخل مراکز چاپ و تکثیر به صورت معناداری بیشتر از فضای آزاد بود (05/0P<). بحث و نتیجه­ گیری: هر چند مراکز چاپ و تکثیر نمی توانند بعنوان یک کانون جدی انتشار آلاینده ها برای سایر اماکن عمومی اطراف خود باشند، اما آلاینده های حاصل از آنها بخصوص آلاینده  O3 می تواند سلامت کارکنان این مراکز را تهدید نماید.

Title: مشکلات روانی اجتماعی والدین کودکان معلول ذهنی تحت پوشش مرکز بهزیستی خرم‌آباد 1392

http://yafte.lums.ac.ir/article-1-2353-fa.html

مقدمه: کودک معلول ذهنی، خانواده را در معرض مشکلات زیادی قرار داده که بیشتر بر روی والدین احساس می‌گردد. لذا درک و شناسایی صحیح از مشکلات و عوامل مرتبط با آن جهت یاری و حمایت آن‌ها ضروری است. در این راستا این مطالعه باهدف تعیین مشکلات روانی اجتماعی والدین کودکان عقب‌مانده ذهنی تحت پوشش مرکز بهزیستی خرم‌آباد انجام گرفت. مواد و روش­ ها: این مطالعه از نوع توصیفی تحلیلی مقطعی بود که نمونه‌های ما را والدین کودکان عقب‌مانده ذهنی تشکیل می‌دادند. ابزار گردآوری داده‌ها پرسشنامه اطلاعات جمعیت شناختی، مشکلات روانی و مشکلات اجتماعی بود. ۱۴۴ پرسشنامه توسط والدین به روش مصاحبه و در منزل تکمیل گردید، روایی و پایایی با استفاده از اعتبار محتوا کسب و اطلاعات  با ۱۶ SPSS  تجزیه ‌و تحلیل شدند. یافته­ ها: نتایج نشان داد که کلیه والدین دارای مشکلات روانی و اجتماعی بودند؛ اما مادران به علت تعدد نقش‌ها در خانواده از میانگین مشکلات بیشتری (مشکلات اجتماعی مادر ۶۹/0±54/1، مشکلات روانی 55/0±46/1 و مشکلات اجتماعی و روانی پدر 74/0±43/1، 55/0±36/1) برخوردار بودند. بین تحصیلات والدین و جنس فرزند معلول ارتباط معنادار (05/0 P<) مشاهده شد. بحث و نتیجه ­گیری: تأثیر معلولیت بر والدین بستگی به توان و ظرفیت آن‌ها دارد. به نظر می‌رسد جهت کاهش مشکلات والدین دارای کودک معلول ذهنی، نیاز به حمایت‌های همه‌جانبه هست.

Title: فیوژن B3A2 شایع‌ترین نوع موتاسیون ABL-BCR در بیماران مبتلا به لوسمی میلوئیدی مزمن در استان لرستان

http://yafte.lums.ac.ir/article-1-2354-fa.html

مقدمه: لوسمی میلوئیدی مزمن (CML) زمانی تشخیص داده می‌شود که وجود کروموزوم فیلادلفیا و فیوژن های ABL-BCR در بیماران اثبات شود. در این مطالعه 58 بیمار مبتلا به لوسمی میلوئیدی مزمن از نظر شیوع انواع فیوژن های ABL-BCR مورد بررسی قرار گرفتند. مواد و روش­ ها: پس از تکمیل پرسشنامه اطلاعات پایه، نمونه خون بیماران با کسب رضایت آگاهانه گرفته شد. RNA از نمونه خون استخراج شد. با استفاده از سنتز cDNA و روش Multiplex RT-PCR فیوژن های مختلف ABL-BCR از جمله فیوژن های b3a2، b2a2 و e1a2 مورد مطالعه قرار گرفتند. یافته ­ها: از میان 58 بیمار که از نظر فیوژن های ABL-BCR مثبت بودند، 18 نفر (5/30 درصد) فیوژن b2a2، 37 نفر (71/62 درصد) فیوژن b3a2 و سه نفر (08/3 درصد) فیوژن e1a2 را دارا بودند. 25 مرد و 33 زن در این مطالعه حضور داشتند که برخلاف مطالعات مشابه، جمعیت زنان مبتلا به CML از مردان مبتلا بیشتر بود. بحث و نتیجه ­گیری: فیوژن b3a2 که تولیدکننده‌ی پروتئینی 210 کیلو دالتونی است، دارای بیشترین شیوع در بین بیماران مورد مطالعه بود. نتایج این مطالعه در استان لرستان (به استثنای جمعیت بیشتر زنان مبتلا) با سایر مطالعات انجام ‌شده در ایران مطابقت داشت.

Title: شیوع آنتی‌بادی‌های ضد سایتومگالو ویروس انسانی در بیماران مبتلا به AIDS/ HIV استان کردستان در سال 1394

http://yafte.lums.ac.ir/article-1-2356-fa.html

مقدمه: عفونت سایتومگالوویروس ((CMVیکی از مهم‌ترین عوامل مرگ و میر و بیماری در افراد دارای نقص ایمنی، همانند دریافت‌کنندگان عضو یا بافت، بیماران مبتلا به HIV و نوزادان تازه متولد شده می باشد. مطالعه حاضر به‌منظور بررسی شیوع آنتی‌بادی‌های CMV در بیماران مبتلا به HIV در استان کردستان انجام گردید. مواد و روش­ ها: مطالعه حاضر به ‌صورت مقطعی روی 151 نفر از مبتلایان به HIV تحت پوشش مراکز و پایگاه‌های مشاوره بیماری‌های رفتاری استان کردستان در سال 1394 انجام گرفت. نمونه سرم بیماران از نظر آنتی‌بادی‌های اختصاصی ضدCMV  با تکنیک الایزا (ELISA)  و کیت تشخیصی رادیم بررسی شد. داده‌ها با استفاده از نرم‌افزار stata   ورژن 13 و با استفاده از آزمون‌های t  مستقل و رگرسیون خطی ساده و چندگانه تحلیل گردید. یافته­ ها:  116 نفر (8/76%) از مبتلایان مرد و 35 نفر (2/23%) زن بودند. میانگین و انحراف معیار سن بیماران به ترتیب 3/39 و 1/9 سال بود. از نظر شیوع سرمی آنتی‌بادی‌های ضد CMV، تمامی بیماران بررسی‌شده از نظر IgG مثبت بودند و تنها یک مورد (7/0%) از نظر IgM مثبت بود. بین سن بیماران و مقدار آنتی‌بادی CMV رابطه معنی‌دار آماری وجود داشت. بحث و نتیجه­ گیری: نتایج مطالعه حاضر نشان‌دهنده شیوع بالای CMV در بیماران مبتلا به HIV بود. بنابراین در کنار توجه بیشتر به بحث عفونت همزمان HIV و CMV، در مورد لزوم و ارزش انجام درمان ضد رتروویروسی زودتر از موقع انجام مطالعات بیشتر ضروری به نظر می‌رسد.

Title: بررسی اثرات ضد باکتریایی مشتقات جدید تیازول، تیازولیدین، ایمیدازول، تتراهیدروپیریمیدین، اکسازولیدین و تیازپین برعلیه انتروکوکوس فکالیس

http://yafte.lums.ac.ir/article-1-2357-fa.html

مقدمه: مقاوم شدن انتروکوکوس فکالیس در برابر طیف وسیعی از آنتی بیوتیک‌ها، به یکی از نگرانی‌های اصلی بخش بهداشت و درمان در اکثر جوامع مبدل شده است و سبب شده است تا بسیاری از محققین شناسایی و استفاده از ترکیبات ضد باکتریایی جدید بر علیه انتروکوکوس فکالیس را به عنوان یکی از مهمترین راه‌کارهای کنترل این پاتوژن مطرح کنند. در این تحقیق به بررسی اثرات ضد باکتریایی 30 مشتق جدید از خانواده‌های تیازول، تیازولیدین، ایمیدازول، تتراهیدروپیریمیدین، اکسازولیدین و تیازپین بر این باکتری پرداخته‌ایم. مواد و روش ها: پس از تهیه مشتقات به صورت محلول در DMSO، جهت بررسی اثرات ضد باکتریایی این ترکیبات از روش انتشار در دیسک جهت اندازه گیری قطر هاله مهار رشد، از روش براث میکرو دایلوشن جهت تعیین حداقل غلظت بازدارندگی رشد (MIC) و همچنین از روش تعیین حداقل غلظت کشندگی (MBC) در مقایسه با آنتی بیوتیک های جنتامایسین و پنی‌سیلین استفاده گردید. یافته ­ها:  پس از تهیه مشتقات به صورت محلول در DMSO، جهت بررسی اثرات ضد باکتریایی این ترکیبات از روش انتشار در دیسک جهت اندازه گیری قطر هاله مهار رشد، از روش براث میکرو دایلوشن جهت تعیین حداقل غلظت بازدارندگی رشد (MIC) و همچنین از روش تعیین حداقل غلظت کشندگی (MBC) در مقایسه با آنتی بیوتیک های جنتامایسین و پنی‌سیلین استفاده گردید. بحث و نتیجه­ گیری: در این مطالعه اثر ضد باکتریایی مشتق جدید ایمیدازول بر روی سویه استاندارد انتروکوکوس فکالیس نشان داده شد و بررسی اثر این مشتق روی سویه‌های مقاوم این پاتوژن، گام بعدی برای شناسایی بیشتر این ترکیب می‌باشد.

Title: بررسی نقش واسطه ای دشواری تنظیم هیجان در رابطه میان مواجهه با وقایع آسیب زا و رفتار پرخطر جنسی در سوء مصرف کنندگان مواد

http://yafte.lums.ac.ir/article-1-2361-fa.html

مقدمه: بررسی‌های انجام شده توسط مرکز مدیریت بیماری‌های وزارت بهداشت نشان می‌دهد موارد ابتلا به ایدز از طریق رفتارهای پر خطر جنسی رو به افزایش است که می‌تواند زنگ خطری برای تشدید عواقب ناگوار این بیماری در جامعه باشد. دشواری تنظیم هیجان و مواجهه با واقعه آسیب زا از مهم‌ترین عوامل دخیل در رفتار پرخطر جنسی هستند. هدف از پژوهش حاضر بررسی نقش واسطه‌ای دشواری تنظیم هیجان در رابطه رفتار پر خطر جنسی و مواجهه با واقعه آسیب زا بود. مواد و روش ­ها: 200 نفر از سوء مصرف کنندگان مواد مراجعه کننده به مراکز درمان و بازپروری اعتیاد در شیراز با روش نمونه گیری در دسترس برای شرکت در پژوهش انتخاب شدند. برای جمع آوری داده‌ها از پرسشنامه رفتارهای پرخطر مربوط به HIV، چک لیست وقایع زندگی و مقیاس مشکلات در نظم بخشی هیجانی استفاده شد. با استفاده از مدل معادله ساختاری مدل علّی احتمالی تحلیل شد. یافته ­ها: نتایج پژوهش از نقش واسطه گری دشواری تنظیم هیجان در رابطه مواجهه با واقعه آسیب زا و رفتار پرخطر جنسی حمایت می‌کند (0001/0 P=،31/0=β). بحث و نتیجه­ گیری: شواهد نشان می‌دهد که مواجهه با وقایع آسیب زا و دشواری تنظیم هیجان می‌توانند زمینه ایجاد رفتار پر خطر جنسی را فراهم کنند. به بیان دیگر واقعه آسیب زا با اختلال در تنظیم هیجانات منجر به رفتار پر خطر می‌شود.

Title: بررسی وجود فاکتورهای ویرولانس در سالمونلا انتریتیدیس جدا شده از نمونه‌های غذایی به ‌روش ‌Multiplex-PCR

http://yafte.lums.ac.ir/article-1-2363-fa.html

مقدمه: سالمونلا انتریکا سروتایپ انتریتیدیس یکی از مهم‌ترین عوامل مسبب سالمونلوزیس غیرتیفوئیدی می‌باشد که از نظر بالینی ‏شدت کمتری نسبت به تب تیفوئیدی دارد. هدف از مطالعه حاضر شناسایی ژن‌های ویرولانس در سویه‌های سالمونلا انتریتیدیس ‏جدا شده از نمونه‌های غذایی به روش ‏Multiplex-PCR‏ می‌باشد.‏ مواد و روش ­ها: این مطالعه توصیفی- مقطعی در سال 1393 بر روی 1250 نمونه غیرتکراری غذایی انجام گردید. روش PCR چندگانه به‌منظور شناسایی ژن‌های invA،ttrC، mgtC، spi4D و agfA انجام شد. یافته ­ها: در مجموع تعداد 60 سویه سالمونلا انتریتیدیس به ترتیب 37 سویه از گوشت مرغ (6/61%) و 23 سویه از تخم مرغ (4/38%) بدست آمد. بیشترین و کمترین فراوانی متعلق به ژن‌های mgtC و spi4D و برابر 6/51% و 3/1% می‌باشد. بحث و نتیجه ­گیری:ب بررسی ژن‌های حدت در سالمونلا انتریتیدیس جدا شده در نمونه‌های غذایی به دلیل فراوانی شاخص‌های ویرولانس و کارایی روش M-PCR در بررسی‌های اپیدمیولوژی و ارزیابی انتقال بین گونه‌ای این ژن‌ها در بین نمونه‌های مختلف می­تواند مفید باشد.

Title: تأثیر مصرف آب انار بدون قند افزوده شده بر روی کنترل گلیسمی، میزان چربی خون و شاخصهای آنتی اکسیدانی افراد مبتلا به دیابت نوع 2

http://yafte.lums.ac.ir/article-1-2366-fa.html

مقدمه: دیابت شیرین از بیماری‌های متابولیک شایع در جهان است. مطالعات نشان داده است که میوه‌ها و سبزیجات و نوشیدنی‌های غنی از ترکیبات فنولی و آنتی اکسیدانی احتمالاً میزان ابتلا به بیماری قلبی-عروقی و دیابت را کاهش می‌دهند. یکی از میوه‌های غنی از فلاونویید، انار است. این مطالعه با هدف ارزیابی تأثیر فرآورده تجاری بدون شیرین کننده آب انار بر روی الگوی قند، لیپید، شاخص‌های تن سنجی و آنتی اکسیدانی و فشار خون انجام شد. مواد و روش ­ها: در این کارآزمایی بالینی،50 بیمار مبتلا به دیابت نوع 2 در محدوده سنی 50-30 سال به‌طور تصادفی به دو گروه تقسیم شدند. گروه مداخله (25 نفر) روزانه 240 سی‌سی آب انار شیرین نشده و گروه کنترل (25 نفر) که روزانه 240 سی‌سی آب مصرف کردند. شاخص‌های تن سنجی، الگوی قندی و لیپیدی، فاکتورهای آنتی اکسیدانی و فشار سیستولی و دیاستولی در قبل و پس از هشت هفته مداخله ارزیابی گردید. یافته­ ها: در ابتدا افراد از نظر نمایه توده بدنی، فشار خون، قند خون، الگوی لیپیدی تفاوت معنی داری نداشتند. در پایان هفته هشتم، در گروه مداخله شاخص مقاومت انسولینی، فشار خون سیستولی و دیاستولی، وزن بدن، دور کمر و دور باسن به‌صورت معنی دار کاهش و غلظت گلوتاتیون پراکسیداز به‌صورت معنی دار افزایش یافت. سطح قند خون، هموگلوبین گلیکوزیله، کلسترول تام، لیپوپروتئین با دانسیته پایین و ظرفیت تام آنتی اکسیدانی در گروه مداخله تغییر معنی داری نیافت. آب انار مانع از کاهش معنی دار سطح لیپوپروتئین با دانسیته بالا شد. بحث و نتیجه ­گیری: نتایج مطالعه حاضر حاکی از تأثیر مثبت آب انار بر بهبود مقاومت انسولینی، فشار خون، وزن، دور کمر و دور باسن و غلظت گلوتاتیون پراکسیداز و سطح لیپوپروتئین با دانسیته بالا بود.

Title: شاخص توده بدنی: مقایسه تنظیم هیجان و رفتار خوردن

http://yafte.lums.ac.ir/article-1-2368-fa.html

مقدمه: چاقی یک مشکل شایع برای سلامت عمومی است که با خطر ایجاد بیماری‌های مختلف همراه است. به‌منظور بهبود درمان و مداخله مناسب در برابر موج فزاینده چاقی، شناسایی عوامل قابل توجه استعداد ابتلا به‌اضافه وزن و چاقی دارای اهمیت حیاتی است. بر همین اساس در این مطالعه به بررسی مقایسه‌ای تنظیم هیجان و رفتار خوردن در زنان با شاخص توده بدنی بالا و طبیعی پرداخته‌ایم. مواد و روش­ ها: مطالعه حاضر از نوع علّی- مقایسه ای بوده، جامعه آماری شامل زنان مراجعه کننده به بیمارستان‌ها و کلینیک‌های تغذیه مناطق یک و دو شهر تهران و زنان با وزن طبیعی ساکن در این مناطق بودند که با روش نمونه گیری در دسترس انتخاب شدند و سپس پرسشنامه‌های دشواری در تنظیم هیجان (DERS) و رفتار خوردن (TFEQ) را تکمیل کردند. داده‌ها با روش آماری t مستقل مورد تجزیه و تحلیل قرار گرفت.  یافته­ ها: نتایج نشان داد عدم پذیرش پاسخ‌های هیجانی، دشواری دست زدن به رفتار هدفمند، دشواری کنترل تکانه، دسترسی محدود به راهبردهای تنظیم هیجان و نمره کل تنظیم هیجان و نیز خوردن هیجانی و خوردن کنترل نشده در افراد چاق به‌طور معناداری بیشتر از افراد با وزن طبیعی است. برعکس خویشتن داری شناختی در افراد چاق به‌طور معناداری کمتر از افراد با وزن طبیعی است.  بحث و نتیجه­ گیری: دشواری‌ در تنظیم هیجان و رفتار خوردن می‌تواند نقش مهمی را در چاقی داشته باشند که نیازمند توجه بیشتر است.
